# Two New Secreted Proteases Generate a Casein-Derived Antimicrobial Peptide in *Bacillus cereus* Food Born Isolate Leading to Bacterial Competition in Milk

**DOI:** 10.3389/fmicb.2018.01148

**Published:** 2018-06-04

**Authors:** Awatef Ouertani, Ines Chaabouni, Amor Mosbah, Justine Long, Mohamed Barakat, Pascal Mansuelle, Olfa Mghirbi, Afef Najjari, Hadda-Imene Ouzari, Ahmed S. Masmoudi, Marc Maresca, Philippe Ortet, Didier Gigmes, Kamel Mabrouk, Ameur Cherif

**Affiliations:** ^1^Université de la Manouba, ISBST, BVBGR-LR11ES31, Biotechpole Sidi Thabet, Ariana, Tunisia; ^2^Aix Marseille University, Centre National de la Recherche Scientifique, ICR UMR 7273, Marseille, France; ^3^Aix-Marseille University, CEA, Centre National de la Recherche Scientifique, LEMiRE, UMR 7265, BIAM, Saint-Paul-lez-Durance, France; ^4^Aix Marseille Univ, Centre National de la Recherche Scientifique, IMM, Plate-Forme Protéomique, MaP IBiSA Labelled, Marseille, France; ^5^Université Tunis El Manar, FST, LMBA (LR03ES03), Campus Universitaire, Tunis, Tunisia; ^6^Aix-Marseille University, Centre National de la Recherche Scientifique, Centrale Marseille, iSm2, Marseille, France

**Keywords:** *B. cereus*, β-casein, antimicrobial peptide, endoproteases, genome sequencing, 3D structure prediction, molecular docking

## Abstract

Milk and dairy products harbor a wide variety of bacterial species that compete for both limited resources and space. Under these competitive conditions, bacteria develop specialized mechanisms to protect themselves during niche colonization and nutrient acquisition processes. The bacterial antagonism mechanisms include the production of antimicrobial agents or molecules that facilitate competitor dispersal. In the present work, a bacterial strain designated RC6 was isolated from Ricotta and identified as *Bacillus cereus*. It generates antimicrobial peptide (AMP) when grown in the presence of casein. The AMP was active against several species of *Bacillus* and *Listeria monocytogenes*. MALDI-TOF analysis of the RP-HPLC purified fractions and amino acid sequencing revealed a molecular mass of 751 Da comprised of a 6-residue sequence, YPVEPF. BLAST analysis showed that the AMP corresponds to the fractions 114–119 of bovine β-casein and represents the product of a specific proteolysis. Analysis of the purified proteolytic fractions from the *B. cereus* RC6 culture supernatant indicated that the presence of at least two different endoproteases is crucial for the generation of the AMP. Indeed, we were able to identify two new candidate endoproteases by means of genome sequencing and functional assignment using a 3D structural model and molecular docking of misannotated hypothetical proteins. In this light, the capacity of *B. cereus* RC6 to generate antimicrobial peptides from casein, through the production of extracellular enzymes, presents a new model of antagonistic competition leading to niche colonization. Hence, as a dairy product contaminant, this strategy may enable proteolytic *B. cereus* RC6 niche specialization in milk matrices.

## Introduction

Most natural environments harbor a wide variety of microbial species (Hibbing et al., 2010). Therefore, bacteria compete with their neighbors for limited resources and niche space (Keymer et al., 2006; Ghoul and Mitri, 2016). The outcome of this interaction is the evolution of a diverse and powerful arsenal of biological arms. Indeed, research into interspecies competitive strategies has revealed that there are diverse mechanisms by which bacterial species can coexist with, or dominate, other organisms competing for the same pool of resources (Stubbendieck et al., 2016). Most bacterial species produce one or numerous potent biocontrol agents, including antibiotics, lytic agents, lysozymes, biofilms, bacteriocins, extracellular vesicles, and enzymes (Riley and Wertz, 2002; Cascales et al., 2007; Nadell et al., 2009; Stubbendieck and Straight, 2016).

To date, several studies have focused on dairy environments. The high nutrient content of dairy products makes them a particularly good growth media for a variety of microorganisms. It is generally accepted that lactic acid bacteria (LAB), such as *Lactobacillus* and *Leuconostoc*, are the dominant fermentative population in milk and dairy products (Quigley et al., 2013). However, psychotrophic microorganisms, yeast and coliform bacteria (although they are not part of the natural microbial population), are found in milk and cheese as spoilage microbes (SamarŽija and Pogačić, 2012). Raw milk is the usual source of spore-forming bacteria in finished dairy products (Ledenbach and Marshall, 2009; Quigley et al., 2013); the most encountered being *B. cereus, B. licheniformis, B. subtilis, B. mycoides*, and *B. megaterium*. *B. cereus* has been isolated from raw milk, pasteurized milk, and cheddar cheese (Ahmed et al., 1983), while dried milk products are also known to be frequently contaminated with *B. cereus* (Becker et al., 1994).

*B. cereus* is a Gram-positive, spore-forming, motile bacterium that can be found ubiquitously (Abriouel et al., 2011) due to its ability to form thermo-resistant endospores, biofilms and to grow over a broad temperature range (Setlow and Setlow, 1994). It is also frequently present in food production environments as a contaminant, due to the adhesive nature of its endospores (Bottone, 2010). Under favorable conditions, spores germinate with the production of a vegetative bacillus, which can then sporulate, maintaining the life cycle (Ceuppens et al., 2013). *B. cereus* is also an opportunistic pathogen that secrets a multitude of pathogenic factors such as collagenase, phospholipases, emetic toxin, enterotoxins, and hemolysins (Gohar et al., 2005; Kilcullen et al., 2016). The contamination of food by *B. cereus* cause spoilage and economical loss (Kumari and Sarkar, 2016). Therefore, ingestion of *B. cereus* by human can lead to two types of disease syndromes: emetic and diarrheal (Tewari and Abdullah, 2015). These syndromes are caused respectively by cereulide and several types of toxins (Carlin et al., 2010). In addition to human intoxication cereulide and toxins play supplementary environmental roles (Ceuppens et al., 2013). They are used as arms during the competition with other microorganisms for resources and space, which increase *B. cereus* fitness during the growth in microbial soil population (Tempelaars et al., 2011). Also, they stimulate the growth of certain plants by inhibition the proliferation of plant pathogen in its rhizosphere (Bullied et al., 2002). Another feature of *B. cereus* is its high-level production of a multitude of metabolites, including bacteriocins, autolysis, and enzymes, particularly proteases (Bizani et al., 2005; Raddadi et al., 2005; Cherif et al., 2007; Chaabouni et al., 2015; Majed et al., 2016). In dairy environments, the proteinases of the contaminating bacteria destabilize casein, the major milk proteins, through hydrolysis. This results in the formation of a gel structure or coagulation of sterilized milk during storage and spoilage (Ledenbach and Marshall, 2009; Majed et al., 2016).

Peptides exhibiting antimicrobial activity have also been identified in hydrolysates of different fractions of casein (Lahov and Regelson, 1996; Haque and Chand, 2008) and exhibit an antibiotic-like activity against a wide variety of microbes (*Staphylococcus* spp., *Sarcina* spp., *Lactobacillus* spp., *B. subtilis, Streptococcus pneumoniae, S. pyogenes, Candida albicans*, and *L. monocytogenes*). Furthermore, peptides released during casein degradation, with different digestive enzymes, have demonstrated *in vivo* antibiotic-like activity, while *in vitro* these peptides were found to inhibit the growth of *Lactobacillus* bacteria and other Gram-positive and Gram-negative bacteria (Dabrowska et al., 2013). Several other studies have shown that several peptides generated through bacterial enzymatic degradation of casein may also play an antimicrobial role (Hayes et al., 2006; López-Expósito et al., 2007; Elbarbary et al., 2012; Atanasova and Ivanova, 2014). In the complex environment of fermented milk products, the production of these degradative enzymes constitutes a prerequisite to obtaining access to nutrients, which leads to balanced microbial communities where competing species coexist. In cases where degradation products exhibit antagonistic activities, the proteolytic bacterial species may benefit from an additional ecological advantage to thrive and to reinforce its niche colonization.

In the present study, we report the identification of two enzymes secreted by a *B. cereus* RC6 strain that permits the degradation of casein, resulting in the generation of an AMP that was identified and characterized. This strategy allows *B. cereus* RC6 to gain a competitive advantage as a contaminant and to thrive in complex ecosystems. Thus, caseinase activity may be an indicator that *B. cereus* RC6 gains a survival advantage in dairy environments shared with competitors. The capacity to release enzymes leading to the generation of an AMP from casein plays a key role in niche colonization within a polymicrobial environment.

## Materials and methods

### Bacterial strains and media

*B. cereus* RC6 was isolated from a Tunisian dairy product “Ricotta” using a selective medium for *B. cereus* agar containing yellow of egg, polymyxin, and mannitol (Chaabouni et al., 2015). *B. thuringiensis* USDA HD22 was used as indicator strain. Tryptic Soy Agar medium (for solid plates) and minimum medium (for broth preparation) were used for detection and production of the antimicrobial product. The minimum medium was composed of (g/l): Na_2_ HPO_4_ 2H_2_O (7.9), KH_2_PO_4_ (3), NaCl (0.5), NH_4_Cl (1), glucose (5), yeast extract (5), and agar (15) for solidification. This minimum medium was adjusted to pH 7.

### Bacterial identification using 16S rRNA gene sequencing

In order to identify the producer strain, DNA was extracted from bacteria and the 16S rRNA gene was amplified by PCR using universal primers as described by Hamdi et al. (2013). The obtained PCR product was sequenced and the 16S rRNA gene sequence of the isolates was compared through a BLAST search (Altschul et al., 1990) with 16S rRNA gene sequences available at the National Centre for Biotechnology Information (NCBI) database (http://www.ncbi.nlm.nih.gov/).

### Characterization of the antimicrobial activity

To evaluate the antimicrobial capacity and to detect if the antimicrobial compound was produced by the bacterium as a secreted metabolite or as the result of casein degradation, *B. cereus* RC6 strain was propagated separately in two different media. The first was a minimum medium with 5% glucose and yeast extract as a carbon source while the second contained 5% glucose and casein. Cell-pellets were removed after centrifugation at 9,000 g for 20 min. Supernatants were subjected to ultrafiltration using Amicon cells with a 10 kDa cut-off, in order to separate peptides of low molecular weight. Fractions less than 10 kDa were tested for their antimicrobial activities against *Escherichia coli* DH5α*, L. monocytogenes* DISTAM MACa1*, B. cereus* ATCC 11778 (BC45)*, B. thuringiensis* USDA HD22, and *S. aureus* ATCC 6539 using the radial diffusion assay method (Lehrer et al., 1991). In order to identify the nature of the antimicrobial compound, the active supernatant was incubated with proteinase K at a final concentration of 1 mg/ml for 2 h at 37°C, then tested for its antimicrobial activity.

### Detection of caseinase activity

In solid medium, protease activity was revealed by spotting a bacterial colony onto a skim milk agar plate and incubating at 30°C for 18 h. A clear zone of hydrolysis with at least 1mm of diameter around the colony indicated protease secretion. In liquid medium, protease secretion was determined by a well diffusion assay. The strain was grown for 24 h in minimum medium and the cells were subsequently removed by centrifugation (9,000 g, 20 min, 4°C). The supernatant obtained was filtered through a 0.22 μm filter (Millipore) and tested for caseinase activity, after cooling in wells created in the skim milk agar plates.

### Antimicrobial compound purification

The antimicrobial compound from the supernatant of *B. cereus* RC6 culture was purified using the following procedure: (i) Ammonium sulfate precipitation (100% saturation) by gradual incorporation of the salt, stirring for 2 h at 4°C, followed by centrifugation at 9,000 g for 20 min. The precipitate was resuspended in water. (ii) Peptides were separated from the precipitate, resuspended in water by ultra-filtration using a bench scale stirred cell centricon (Amicon Millipore) filter device with a 10 kDa cut-off. Eluted fractions were collected, lyophilized and tested for their antibacterial activity against the indicator strain. (iii) Reverse Phase High Performance Liquid Chromatography (RP-HPLC) purification of the active fraction was performed using a semi preparative C18 column (Chromolith 100–10 mm). Solvent A [Acetonitrile 0.1% Trifluoroacetic acid (TFA)] and solvent B (H_2_O 0.1% TFA) were used as the mobile phase with a flow rate of 6 ml/min. The elution of the different peaks was performed with 60% acetonitrile over 60 min. Active molecule associated-peaks were lyophilized, dissolved in distilled water and tested for antimicrobial activity.

The active peaks selected above were purified a second time and their homogeneity assessed by RP-HPLC coupled to LC/MS on a C18 reverse phase column. Elution was performed using 60% acetonitrile over 60 min with a flow rate of 1 ml/min. The antibacterial activity of each peak was evaluated by the same method as in the first purification step and active peaks were subjected to identification.

### Peptide identification

Absorbance spectra of the purified peptide were measured with a UV 2401 pc Shimadzu spectrophotometer. The purified peptide was diluted using ultrapure water and the absorbance was measured from 200 to 300nm using quartz cuvets. Data were collected and analyzed with the UV 2401 pc Shimadzu software.

LC/MS analysis experiments were performed on a LC/MS Q TOF (Shimadzu Corporation Kyoto Japan) using an ESI ion source, operated under negative ion mode. LC-MS separation was achieved on a C18 Phenomenex 150 × 4mm column at 35°C. The wavelengths of spectrum detection were 214 and 280nm. Elution was carried out with solvent A (Acetonitrile 0.1% TFA) and solvent B (H_2_O 0.1% TFA). A linear gradient from 0 to 60% solvent A was applied over 60min. The injection volume was 20 μl and the flow rate was set to 1 ml/min.

MALDI-TOF mass spectrometry analysis was performed on a Microflex II (Bruker Daltonics). The lyophilized peptide was suspended in water with 0.1% TFA and 1 μl of the solution was mixed with 1 μl of α*-*Cyano*-*4*-*hydroxycinnamic acid (HCCA) matrix. This resulting solution was spotted onto the MALDI 96 well stainless-steel sample plate and allowed to air dry prior to MALDI analysis. Acquisitions were performed in a reflector positive mode, with external calibration, using a standard peptide mixture from Bruker. For amino acid sequencing, the active peptide was deposited on a pre-treated BioBrene filter, dried and placed in cartridge B of the Procise 494A sequencer. A program of 10 cycles of Edman degradation was performed using the “pulsed liquid protein.”

### Identification of the protease activity leading to AMP

The *B. cereus* RC6 strain was propagated in theminimum medium and the supernatant was fractionated using an Amicon Millipore cell with a 10 kDa cut-off. The two obtained fractions [molecular weight (MW) lower and higher than 10 kDa] were tested for their capacity to degrade casein. The active fraction was fractionated by RP-HPLC using a preparative C18 column (250–21 mm, particles 5 μm, pores 100 Å) from MACHERY NAGEL. Acetonitrile 0.1% TFA (solvent B) was used as the mobile phase, with a flow rate of 6 ml/min. The elution was performed with the following gradient: from 0 to 30% solvent B over 60min and from 30 to 100% solvent B over 20min. Fractions were collected every 3min, lyophilized and tested for their proteolytic activity. Fractions showing proteolytic activity were analyzed using an Agilent 2100 bioanalyzer system, according to the manufacturer's procedure (protein 80 kit).

The proteolysis zones of casein degradations in the agar medium were cut off, eluted, dissolved in sterile distilled water and then tested for antimicrobial activity.

### Basic analysis of *B. cereus* RC6 genome sequence

The total genomic DNA of *B. cereus* RC6 was extracted with Analytik Jena DNA and RNA kits following the manufacturer's instruction. Genomic DNA was sequenced using Illumina HiSeq technology, and FASTQ- formatted sequence data was created. The resulting genome was annotated with SPADES and bio-informatically analyzed using both Prodigal and PRIAM base (Claudel-Renard et al., 2003).

### Functions prediction of unknown or hypothetical proteins using structural analysis

The proteins annotated as conserved hypothetical proteins from the whole genome sequence were selected and composed the initial data set of the bioinformatic analysis. The remaining sequences were submitted to SignalP 4.0 (Petersen et al., 2011) to predict transmembrane topology and signal peptide sequences. The predicted transmembrane proteins were discarded, and the signal sequences were removed from the translated sequence, generating a data set of mature and non-transmembrane sequences. A size selection was performed using ExPASy MW. Then, only the small sequences ranging from 85 to 100 amino acid residues were selected. Thus, the functions and structures of these selected proteins were predicted by Iterative Threading ASSEmbly Refinement server (I-TASSER), the online structure prediction tool (Yang and Zhang, 2015) according to the results of protein analysis (Agilent 2100). Template structures were selected by a multiple threading approach with the Local Meta-Threading-Server (LOMETS) based on sequence similarity to the structure described. A total of five three-dimensional structure models were generated by I-TASSER with each model. Among them, the best model was identified based on a confidence score (C-Score), and refined model coordinates were evaluated by MolProbity (Davis et al., 2007). All models were assessed using coach (Yang et al., 2013), a software for protein ligand binding site prediction. The result identified the protein with protease function with a C-score. The best proteases were selected according to their C score. A final analysis with MolProbity was assessed to validate and to analyze the quality of the structures. Seven selected structures were compared to analogous structures in the goal to select their functions.

### Molecular docking

The bovine ß-casein (113–120) fragment sequence was blasted against the PDB database using blastp. The PDB structure of the ß-casein (113–120) fragment was used as the ligand for molecular docking. Modeling of the selected ligand binding to protease and cleavage after Phe was performed with a rigid protein-protein docking software package based on different approaches, namely the PatchDock and FireDock programs (Fast Interaction Refinement in molecular DOCKing: http://bioinfo3d.cs.tau.ac.il/FireDock/) (Andrusier et al., 2007). PSAIA was used to determine the residue interaction pairs and binding residues (Mihel et al., 2008).

### Phylogenetic and phylogenomic analysis

For phylogenomic analysis, the entire genome data from 23 different strains of *B. cereus*, available at NCBI, were downloaded and used for comparative genomics. The previously described method for enzyme annotation was used on each of the 23 genomes and the resulting predicted peptidases (*B. cereus* RC6 P1 and P2) were clustered and compared using CD-HIT (Li and Godzik, 2006).

## Results

### Bacterial strain identification and protease—antimicrobial activity correlation

Analysis of the 16S rDNA gene sequence from the *B. cereus* RC6 strain revealed a 99% identity with *B. cereus* reference strains. The rRNA gene was sequenced and the resulting sequence compared to the NCBI (National Centre for Biotechnology Information Data Base: www.ncbi.nlm.nih.gov) 16S rRNA database using BLAST.

**Figure 1:**
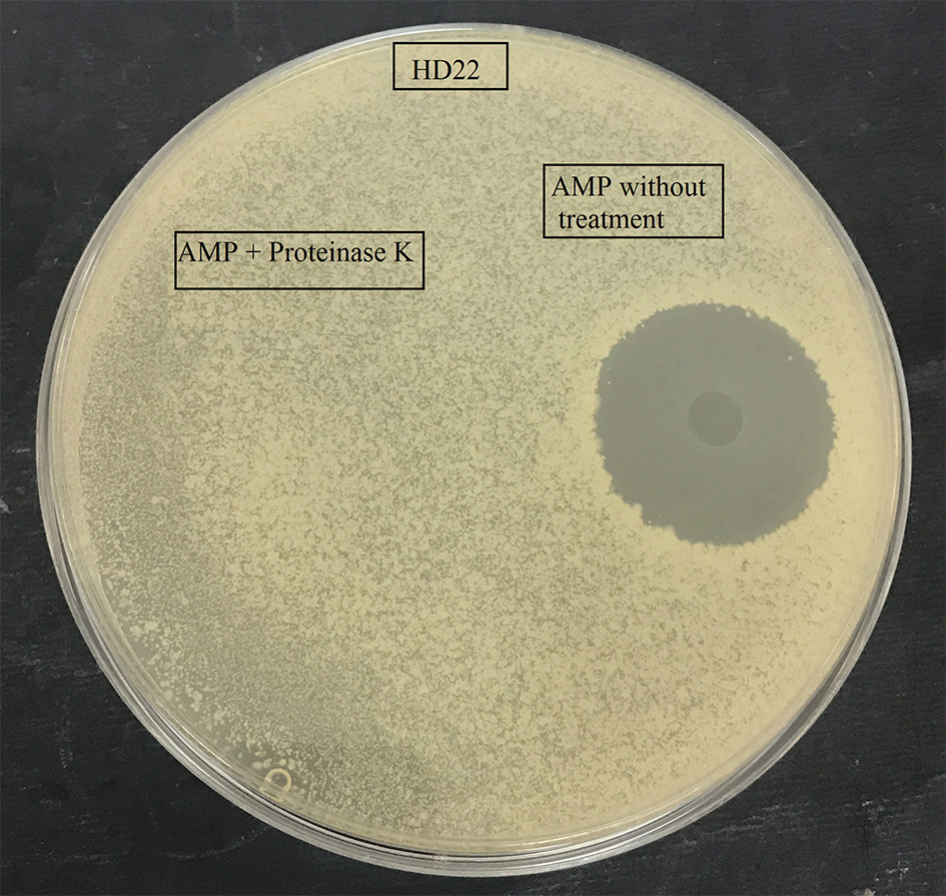
Effect of proteinase K treatment on antimicrobial activity of the purified AMP against *B. thuringiensis* HD22.

Antimicrobial testing showed that the *B. cereus* RC6 strain displays antimicrobial activity only when propagated onminimum medium supplemented with 5% casein. The highest antimicrobial activity was exhibited against closely related bacteria, including *B. cereus* ATCC 11778 (BC45) and *B. thuringiensis* USDA HD22. Antimicrobial activity was also observed against the food pathogen *L. monocytogenes* DISTAM MACa1. However, no activity was observed against *S. aureus* ATCC 6539 and the pathogenic Gram-negative bacteria *E. coli* DH5α (Table 1). This activity against *B. thuringiensis* USDA HD22 was completely lost when treated with proteinase K (Figure 1). These results show, on the one hand, the oligopeptidic nature of the antimicrobial agent and, on the other hand, that the active compound is either secreted by the bacteria in the presence of casein or that it is later generated through hydrolysis. Observation of the *B. cereus* RC6 strain phenotype on skim milk/casein agar medium revealed a clear halo around the colony, indicating protease activity and raising the question of the possible involvement of this activity in casein degradation and AMP generation.

**Table 1:** Antimicrobial spectra of the active supernatant of *B.cereus* RC6.

**Indicator organism**	Inhibition zone(mm)*
*B. thuringiensis* USDA HD22	+++
*B. cereus* ATCC 11778 (BC45)	+++
*L. monocytogenes* DISTAM MACa1	+
*S. aureus* ATCC 6539	–
*Escherichia coli* DH5α	–

* *Activity is expressed as the diameter of the inhibition zone around the well: +, < 10 mm; ++, < 20 mm; +++, < 30 mm; –, no activity*.

### Purification and identification of the antimicrobial peptide

**Figure 2:**
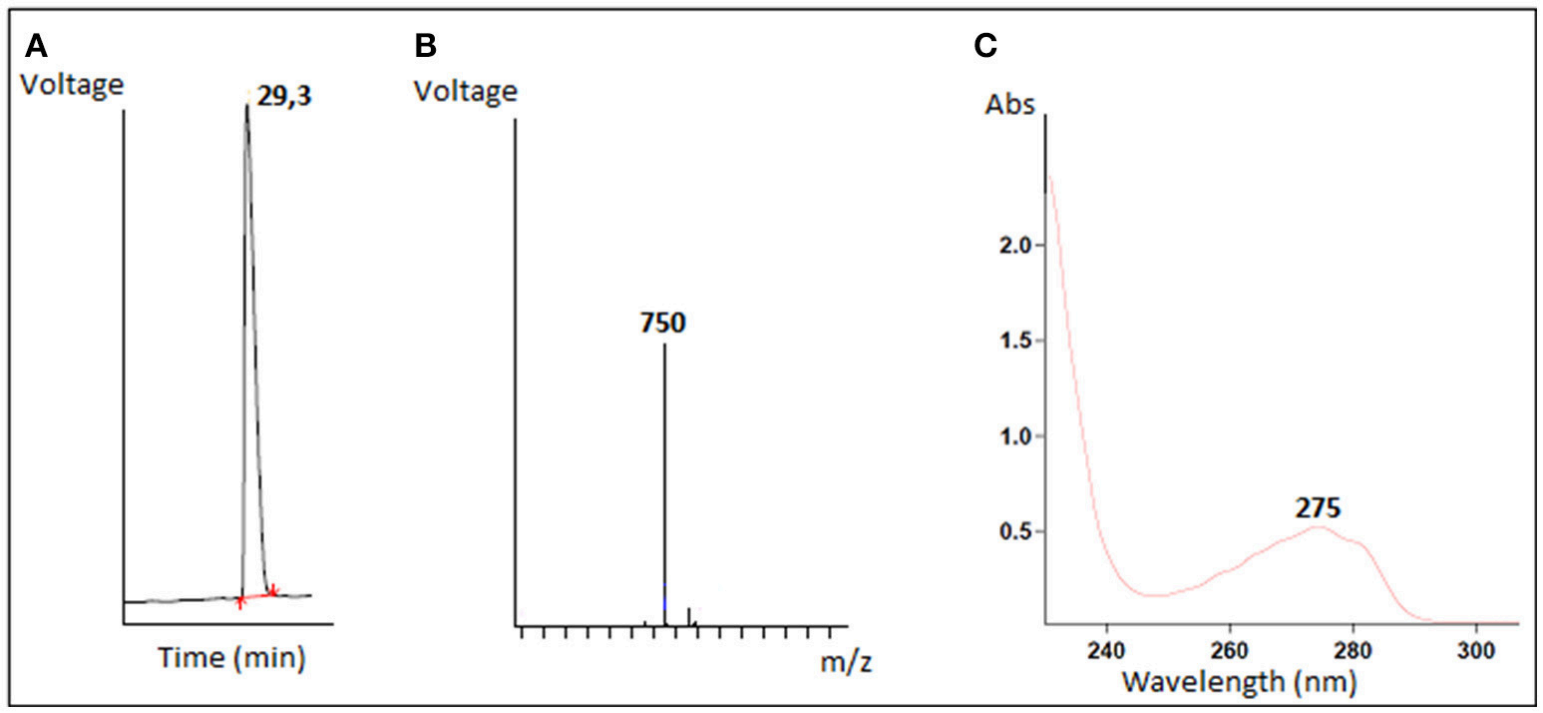
Characterization of purified antimicrobial peptide **(A)** Elution profile of the active fraction on reverse phase HPLC, **(B)** LC/MS mass spectrum, **(C)** The absorbance spectrum scan.

The antimicrobial compound was purified from the *B. cereus* RC6 supernatant by recovering the active antibacterial agent from the filtrate after ultra-filtration through a 10 kDa cut off membrane. This result indicates that the AMP, as demonstrated by its sensitivity to proteinase K, has a molecular mass <10 kDa and is likely peptidic in nature. The antimicrobial molecule was then separated and purified on a semi preparative RP-HPLC. The eluted peaks were collected and analyzed for antibacterial activity. The active fraction was purified by subsequent RP chromatography and the antimicrobial activity was found in the peak eluted around 30min (Figure 2A).

Mass spectrometry analysis revealed an intense major peak corresponding to a molecular mass of 751.3 Da. This result was consistent with the LC/MS analysis which showed a single signal corresponding to 750 Da (Figure 2B). The purified peptide was subjected to amino acid sequencing using Edman degradation. The result revealed a sequence of 6 amino acids (YPVEPF) with a theoretical mass of 750.359 Da as determined by the “sequence editor” software. The measured mass (M + H) is 751.299 Da, or 750.299 Da for M. The excellent correlation between the MALDI-TOF, LC/MS and Edman degradation analyses confirm the sequence was identified in its totality. The blast analysis revealed that the peptide corresponds to the fragment 114–119 of bovine β-casein.

The resulting absorbance spectrum of the purified peptide is characteristic of a polypeptide containing aromatic residues. Furthermore, the observed absorption maximum at 275 nm is indicative of the presence of tyrosine residues (Figure 2C).

### Identification of the protease responsible for the casein degradation

**Figure 3:**
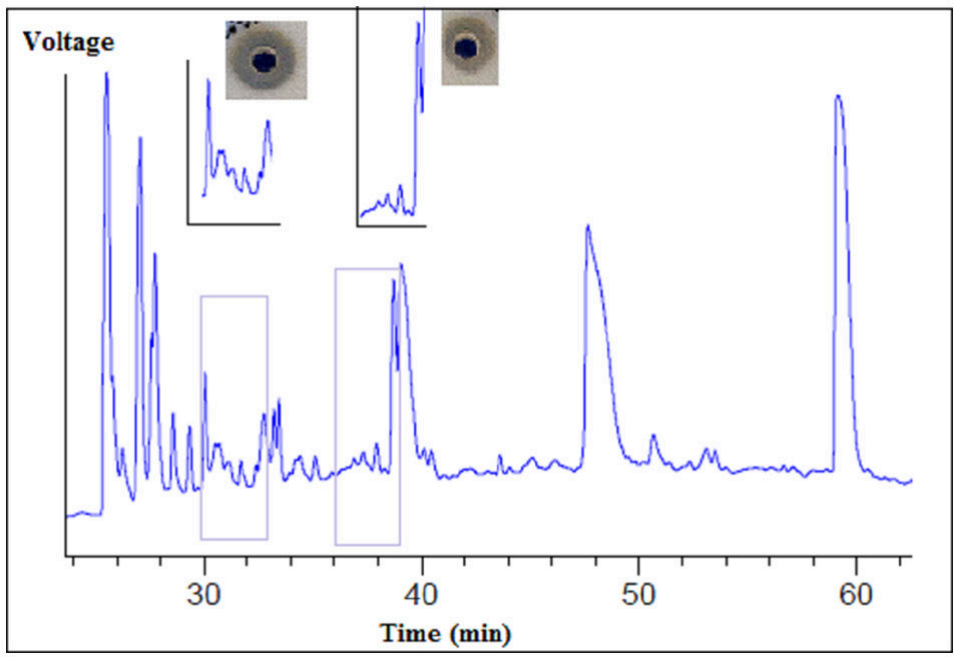
Separation of proteolytic enzymes from *B. cereus* RC6 strain supernatant by using reverse phase HPLC.

The supernatant of the *B. cereus* RC6 strain demonstrated proteolytic activity. After ultrafiltration, only fractions larger than 10 kDa showed this activity. Several fractions were obtained by HPLC separation. After lyophilization, each fraction was analyzed for caseinolytic activity. These analyses revealed that the fractions eluted at the 30–33 and 36–39min retention time intervals exhibit caseinolytic activity (Figure 3).

**Figure 4:**
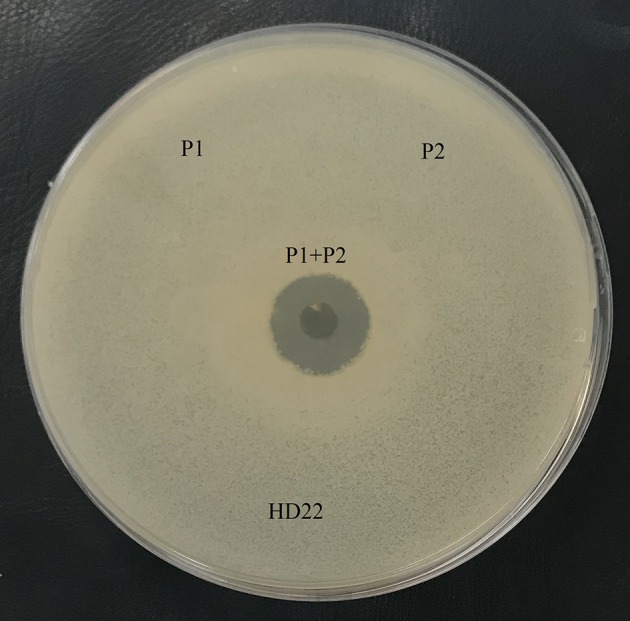
Antimicrobial activity of the fraction with caseinase activity, P1: protease 1 (fraction 30-33), P2: protease 2 (fraction 36–39). *B. thuringiensis* HD22 was used as the indicator strain.

Antibacterial analysis of the agar-eluted fractions from the casein degradation zones revealed that both enzymes are essential to AMP generation. However, no activity was observed when each enzyme was tested separately, and this antibacterial activity appeared only when the two fractions were present (Figure 4).

The fractions demonstrating caseinolytic activity were analyzed using an Agilent 2100 expert-protein-80 bioanalyzer system. This analysis revealed two major peaks with molecular weights of 10.7 and 11.8 kDa for the fractions eluted at the 30–33 and 36–39min retention time intervals, respectively (data not shown).

### Genome analysis and protease prediction

Genomic DNA of the *B. cereus* RC6 strain was sequenced using Illumina HiSeq technology. A total of 23.448.526 paired-end reads were obtained with a mean read length of 125 bases. We compared two publicly available and commonly used de novo genome assembly tools. The first, ABySS (Simpson et al., 2009), was run with the k-mer optimal size set to 64. It assembled 86 scaffolds longer than 500 base pairs (bp), for a total length of 5.9 Mbp. The longest scaffold was 343.440 bp and the N50 parameter was 178.043 bp. The GC content was estimated at 35.17% by QUAST (Gurevich et al., 2013), which provides information on the assemblies and allows for their evaluation (genome analysis to be published).

Spades (Bankevich et al., 2012), the second assembly tool, was applied to assemble the sequencing reads into 101 scaffolds longer than 500 bp for a total genome length of 5.7 Mbp. The longest scaffold was 358.737 bp and the N50 parameter was 172.765 bp. The GC content was estimated at 35.19%.

Gene prediction was accomplished using Prodigal (Hyatt et al., 2010), which identified 5,711 protein-coding genes. We compared the predicted proteome against the MEROPS database (Rawlings et al., 2016), which regroups information about peptidases, their substrates, and inhibitors. The database has been used to identify the proteolytic enzymes implicated in the cleavage of the β-casein protein from *Bos taurus*. Two predicted enzymes with caseinolytic activity were found, however, the cleavage sites of these enzymes did not generate the desired peptide of interest. We subsequently decided to target the set of hypothetical proteins. To this end we functionally annotated all the protein-coding genes and extracted a pool of 1,751 proteins without any established function, using the Swissprot database (The UniProt C, 2017). As the peptide signal should be absent from the mature peptides, their sequences were removed from the analyzed protein sequences and a size cut-off was applied to select those ranging from 85 to 100 amino-acids, according to the protein analysis results obtained. This reduced the number of sequences to 207. Prediction of the proteases able to cleave after Lys and Phe, as well as their 3D structure, were performed by the I-TASSER online server using the template obtained by searching against the Protein Data Bank database. Five models were generated for each protein with C-scores ranging from (−5) to 2 and those having the highest C-score, representing the best models, were selected (7 sequences left).

Ten PDB structures close to the target were generated for the 7 sequences. The structural similarity between the target model and the 10 closest proteins were ranked by TM-scores (Zhang and Skolnick, 2005). Analysis of the PDB structure of the 7 sequences allowed us to identify two proteases, P1 and P2, which could potentially generate the peptide YPVEPF. P1 encoded by the gene bac 4,240 cleaves bovine β-casein after the Lys residue and P2 encoded by the gene bac 3,135 cleaves after the Phe residue.

**Figure 5:**
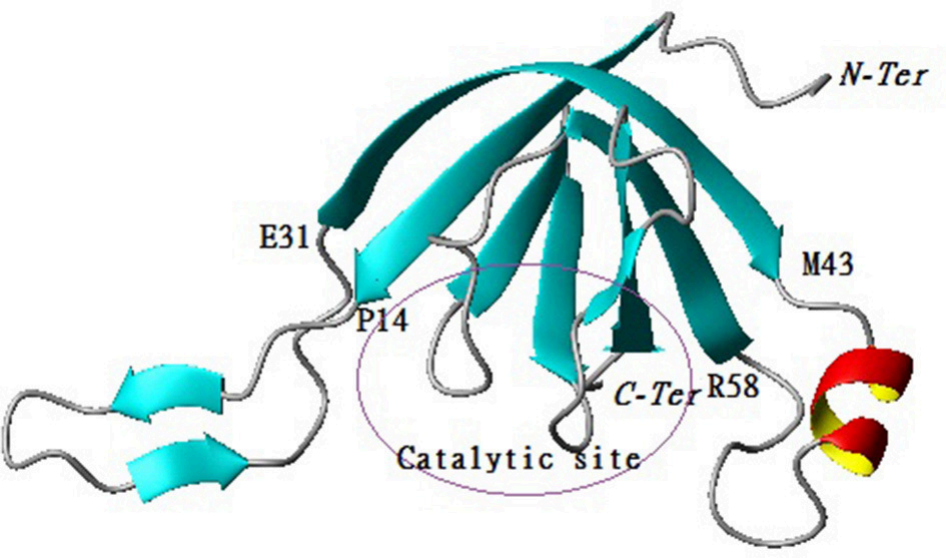
Cartoon representation of 3D structure of protease 1 with the active site localization.

The predicted model for P1 indicates that the protein belongs to the family of serine proteases. In addition, the structural analysis demonstrated that the enzyme on one side consists of a central barrel formed by 4 strands paired in antiparallel ß-sheets (Arg6-Glu13, Val32-Ser38, Arg75-Met78, Gly80-Iso85) while the other side is formed by 3 strands paired in antiparallel ß-sheets (Trp37-Thr42, Val59-Pro65, Thr98-Lys104) with two emerging loops. The first loop (Pro14-Gln31) and the second one contained one ß-sheet (Val16-Lys18, Gln23-Iso25) and one α -helix (Lys44-Glu50) (Figure 5).

**Figure 6:**
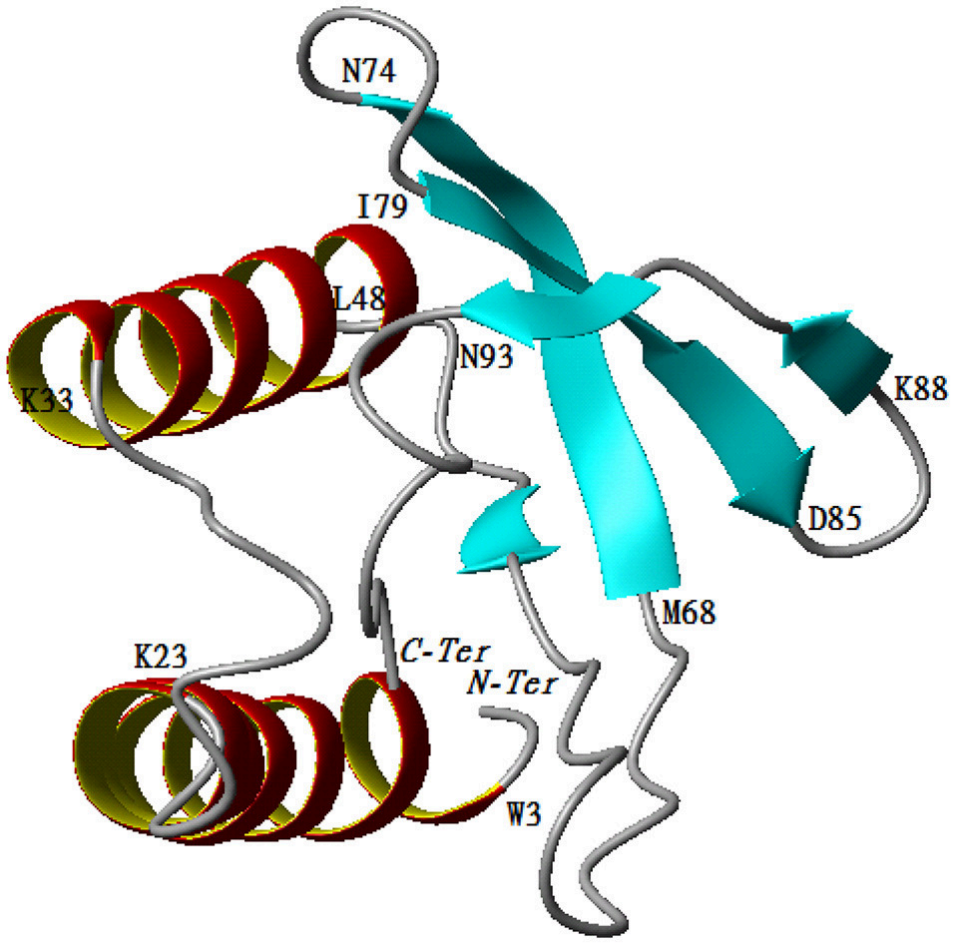
Cartoon representation of 3D structure of protease 2.

The predicted model for P2 indicates that the protein belongs to the family of papain-like proteases. Structural analysis demonstrated that the enzyme was composed of two regions. The first one is a helix turn helix formed by two helices (Trp3-Lys23, Lys33-Leu48). The second one is an antiparallel ß-sheet formed by the following three arms, Met68-Asn74, Iso79-Asp84, and Lys88-Asn93. The two regions are separated by one mobile loop located between Gln50 and Tyr67 that allows for accessibility to the active site. The C-terminal region (Thr 94-Ala101) tends to form a helicoidal region pointing to the active site (Figure 6).

### Molecular docking

**Figure 7:**
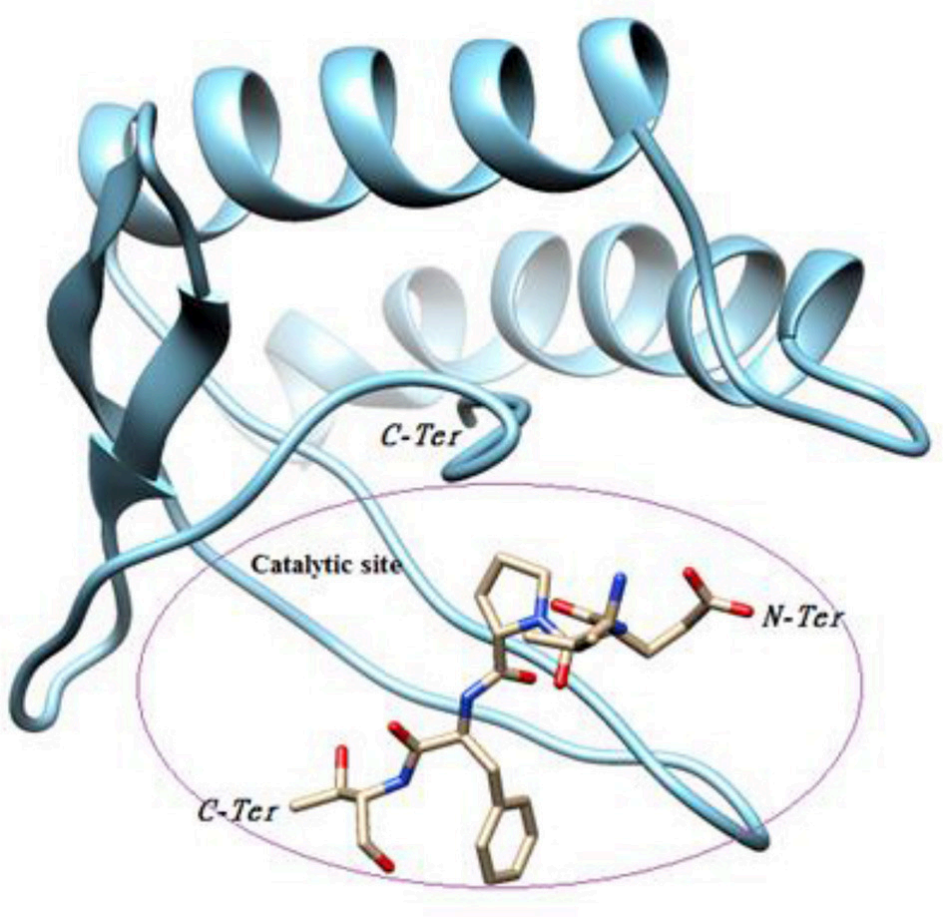
Substrate binding studies on the surface of P2.

Our objective was to identify possible interactions between the catalytic triad (Cys82, Ser46, Asp41) of P2 and the bovine ß-casein (117–120) fragment. Two programs, PatchDock and FireDock, were employed for protein-protein docking using P2 as a receptor and the ß-casein (117–120) peptide as a ligand. A thousand predictions were generated using PatchDock and all were submitted to FireDock to refine the 10 best solutions based on global energy. Several low-energy docking models emerging from this exercise placed the active site of P2 close to the peptide. Amongst these models, one complex was found to be plausible based on theminimum energy score (−89.19) and binding interface residues (Table 2). This model demonstrates that the ligand attachment site is located near the catalytic site (Figure 7). This interaction allows us to confirm the proteolytic activity of P2. Therefore, P2 cleaves the bovine ß-casein (117–120) fragment between Phe119 and Thr120. Subsequent analysis of the residue interaction pairs and binding residues using PSAIA revealed four main types of interactions that enhance the binding of the ligand to the receptor: Van der Waals, ionic, polar, and hydrophobic.

**Table 2:** Binding residues implicated on β-casein and *B. cereus* P2 interaction.

**Ligand**	**Receptor**
Pro	Tyr 57
	Gln 59
	Phe 92
	Asp 96
	Iso 97
Phe	Tyr 57
	Phe 58
	Tyr 62
	Leu 64
	Phe 92
Thr	Tyr 57
	Leu 64

### Phylogenetic and phylogenomic analysis

Clustering (50% identity) and phylogenetic analyses of the 2 proteins (*B. cereus* P1 and P2) were realized by comparison with the 23 *B. cereus* selected genomes. For the *B. cereus* RC6 P1, only 10 proteins from 7 different genomes contained an orthologue. The other genomes did not contain an orthologue of at least 50% identity. The genomes were also clustered at 40% identity, and the same result was obtained. For *B. cereus* RC6 P2, 21 genomes are involved with at least one protein per genome. In all, 25 proteins were identified (for example, *B. cereus* MM3 has 2 proteins; Table 3). All identified proteins displayed low homology with *B. cereus* RC6 P1 and P2 ranging from 62 to 92% for P1 and from 51 to 82% for P2. Analysis of the identified proteins revealed that all were misannotated and had never before been reported as proteases.

**Table 3:** Comparative analysis of *B. cereus* P1 and P2 with some *B. cereus* strains.

**Strain**	**Origin**	***B. cereus*** **RC6 P1 (Spades 4240)**			***B. cereus*** **RC6 P2 (Spades 3135)**		
		**NCBI gene ID**	**Gene function**	**% Identity**	**NCBI gene ID**	**Gene function**	**% Identity**
*B. cereus* NC7401	Soil	–	–	–	BAL18052.1	Hypothetical protein	76.58
*B. cereus* AH621		–	–	–	EEK73390.1	Hypothetical protein	73.87
*B. cereus* ATCC 14579		–	–	–	AAP09239.1	Hypothetical cytosolic protein	81.98
*B. cereus* E33L		–	–	–	AAU18152.1	Hypothetical protein	74.77
					AAY60475.1	Conserved hypothetical protein (plasmid)	53.76
*B. cereus* HuB2-9		–	–	–	EJV93822.1	Hypothetical protein	75
*B. cereus* 03BB87	Clinical	–	–	–	AIY75373.1	YoI-D- like family	78.38
*B. cereus* B06.009		–	–	–	OBZ55822.1	Hypothetical protein	76.58
*B. cereus* 172560W		EEK59187.1	Phage head tail protein	92.31	EEK62299.1	Hypothetical protein	81.08
*B. cereus* R309803		–	–	–	EEK78944.1	Hypothetical protein	73.04
*B. cereus* 95/8201		EEL16940.1	Phage head tail adaptor	93.16	EEL16285.1	Hypothetical protein	51.96
					EEL17160.1	Hypothetical protein	76.58
*B. cereus* m1550	Uncooked chicken	EEK89252.1	Phage head tail adaptor	93.16	EEK89647.1	Hypothetical protein	81.98
*B. cereus* FORC 005	Korean foods	AKE16985.1	Phage protein	84.48	AKE16680.1	Hypothetical protein	81.98
*B. cereus* MM3	Food	–	–	–	EEK64477.1	Hypothetical protein	52.69
					EEK67624.1	Hypothetical protein	80.00
*B. cereus* NVH0597-99	Spice mix	–	–	–	EDX67783.1	Conserved domain protein	76.58
*B. cereus* NZAS01	Food stuffs	–	–	–	OKA30056.1	Hypothetical protein	74.77
*B.cereus* TIAC219	Pasta	EOQ59330.1	Hypothetical protein	80	EOQ64532.1	Hypothetical protein	79.28
		EOQ59789.1	Hypothetical protein	62.50			
		EOQ69234.1	Hypothetical protein	86.21			
		EOQ69788.1	Hypothetical protein	90.60			
*B. cereus* K-5975c	Pasta salad	EOO88683.1	Hypothetical protein	88.89	EOO85243.1	Hypothetical protein	79.28
*B. cereus* 10987	Dairy	–	–	–	AAS41279.1	Conserved hypothetical protein (plasmid)	76.58
					AAS44857.1	Conserved hypothetical protein (plasmid)	52.08
*B. cereus* AH603		EEL69657.1	Phage head tail adaptor	86.21	EEL71004.1	Hypothetical protein	73.87
*B. cereus* m1293		–	–	–	EEK44985.1	Conserved domain	76.00
*B. cereus* FSL K6-0043		–	–	–	–	–	–
*B. cereus* 16SBCL1279		–	–	–	OJS92925.1	Hypothetical protein	81.98

## Discussion

Microbes compete to survive in diverse environments. The ability of bacteria to develop several competitive mechanisms and responses to challenges may be indispensable to their survival in communities of various environments (Stubbendieck and Straight, 2016). The *B. cereus* RC6 strain has been isolated from dairy product (LeBlanc et al., 2002). Indeed, milk and dairy products are favorable environments for the growth of *B. cereus* as a contaminant (Janštová et al., 2006). In general, milk contaminated by *B. cereus* occurs either at the farm or during processing of final products, such as UHT milk (Vidal et al., 2016). Milk proteins exert a wide range of nutritional, functional, and biological activities. Moreover, many milk proteins possess specific biological properties, which make them potential ingredients of health-promoting foods. Therefore, increased attention is being given to active peptides derived from milk proteins. These peptides are inactive within the sequence of the parent protein molecule and can be liberated by (1) gastrointestinal digestion of milk, (2) fermentation of milk with proteolytic starter cultures, or (3) hydrolysis by proteolytic enzymes (Korhonen and Pihlanto, 2006). Indeed, the ability of *B. cereus* to degrade milk proteins, particularly casein, has already been reported (Janštová et al., 2006; Jadhav et al., 2014). Another important property of *Bacillus* spp. is the ability of their vegetative cells to produce thermally stable extracellular enzymes after proliferation (Meer et al., 1991; Ipsen et al., 2000). These enzymes hydrolyze milk proteins, which affect nutritional and sensory properties even if viable bacteria are not present (Boor et al., 1998). Lopez-Fandino et al. (1993) reported that *B. cereus* enzymes first break down casein before they start breaking down whey proteins (Lopez-Fandino et al., 1993). In addition, Melachouris and Tuckey (1968) reported that β-casein was more readily degraded by *B. cereus* protease than other casein fractions and total degradation of β-casein was observed after 40min (Melachouris and Tuckey, 1968). In the present study, we showed that two new proteases from the *B. cereus* RC6 strain degrade bovine β-casein resulting in the generation of an AMP. This AMP inhibits the growth of various Gram-positive bacteria including *B. cereus, B. thuringiensis*, and *L. monocytogenes*. This characteristic is typical of AMPs produced by Gram-positive bacteria which are mostly active against Gram-positive bacteria and are less effective against Gram-negative bacteria (Gray et al., 2006). The AMP was eluted using HPLC with a 29% acetonitrile mobile phase, which indicates a high degree of hydrophobicity. The molecular weight of the peptide obtained by MALDI-TOF was 751 Da and Edman degradation revealed an YPVEPF amino acid sequence. The strong correlation of the molecular weights obtained by MALDI-TOF and LC/MS analysis allow us to confirm that the sequence was determined in its totality. The sequence obtained in the present study revealed the absence of modified amino acids. Remarkably, the peptide amino acid sequence showed a lack of cationic amino acids such as Lys and Arg, which are typically found in AMPs (Huang et al., 2007). This can be explained by the presence of proline and aromatic amino acids, which have been shown to play a critical role in the activity of some AMPs (Bizani et al., 2005). Indeed, previous researchers have reported that the most active antibacterial peptides against pathogens belong to the proline-rich peptide family (Otvos, 2002; Cao et al., 2015). In addition, analysis of the peptide using the PROWL database was in agreement with the BLAST analysis of the amino acid sequence using the GenBank protein database, revealing that the peptide corresponds to the fraction (114–119) of bovine β-casein. Several AMPs generated by bacterial degradation of casein have been reported. For example, *S. thermophilus* strains degrade β and α-casein resulting in the generation of antibacterial peptides from β-casein (f l93–209) and from α-casein (f l−23) (Miclo et al., 2012). Similarly, Hayes et al. (2006) reported that the degradation of α-casein by *L. acidophilus* DPC6026 results in the production of three peptides displaying antibacterial activities against the pathogenic strains *Enterobacter sakazakii* ATCC 12868 and *E. coli* DPC5063 (Hayes et al., 2006). Finally, *Lactococcus lactis* subsp. *lactis* BR16 was found to hydrolyze bovine casein through the secretion of extracellular serine metalloproteases, generating a new antibacterial peptide from αs2-casein corresponding to the fragment (24–31; Bougherraa et al., 2017).

Analysis of the proteolytic activity of different HPLC fractions obtained from the RC6 supernatant demonstrated that the *B. cereus* RC6 strain secretes two new enzymes essential for the generation of an AMP derived from casein. Genome analysis and the prediction of the proteolytic enzymes implicated in the cleavage of the β-casein protein did not allow for the identification of the endoproteases of interest.

Analysis of the 3D structural models and the function of protein annotated as hypothetical, revealed two enzymes implicated in the generation of AMP. The protease that cleaves the upstream sequence was identified as a member of the family of serine protease (EC number 3.4.21.34). Analysis of the PDB structure indicated high Root Mean Square Deviation (RMSD) (3.80) between our new protease (P1) and the Rv3671C serine protease secreted by *Mycobacterium tuberculosis*, which has been implicated in the protection of the producer from oxidative and acidic stress (Biswas et al., 2010). Consequently, our protease may have the same mechanism of action. In fact, like the Rv3671C serine protease, *B. cereus* RC6 P1 protease acts as a clasp by embracing regions containing residues of the catalytic triad (His72, Ser68, and Gln87). The Rv3671C serine protease also displays multiple turnover cleavage of β-casein (Biswas et al., 2010). Furthermore, the homology between the two structures leads us to speculate that the secretion of this enzyme by *B. cereus* RC6 provides an ecological advantage, by protecting producer cells from oxidative and acidic stress. In fact, *B. cereus* RC6 was isolated from a fermented milk product where it cohabits with various fermentative bacteria, such as *Lactobacillus*, which produce lactic acid as a major end product for milk acidification (Tannock, 2004).

The protease that cleaves the downstream sequence was identified as a member of the papain-like cysteine proteases family (EC number 3.4.21.34). Interestingly, analysis of the PDB structure revealed a high RMSD (3.38) between the *B. cereus* RC6 protease P2 and the *Salmonella typhimurium* effector GtgE (Xu et al., 2016). Like GtgE, the protease P2 may play an essential role in virulence (Figueroa-Bossi and Bossi, 1999) and could also allow the producer to overcome the host restriction barrier (Xu et al., 2016). Furthermore, the presence of the protein effector GtgE in *S. typhimurium* enables it to colonize other niches (Spano and Galan, 2012) which could also be the case for the dairy product contaminating *B. cereus* RC6. Molecular docking analyses revealed that the new RC6 P2 protease can bind and cleave the target bovine β-casein (117–120), demonstrating its clear role in AMP generation in milk.

Our finding demonstrates that proteases P1 and P2 are effective in generating the YPVEPF antimicrobial motif from casein. As an ecological attribute, when bacteria use secreted effectors like enzymes, they are able to compete whileminimizing the risks of direct damage during contact-mediated competition (Stubbendieck and Straight, 2016). Thus, the capacity of the *B. cereus* RC6 strain to release enzymes that degrade casein resulting in an AMP is a competitive advantage. Furthermore, enzymes and compounds produced and secreted by bacteria can interfere with quorum sensing. In fact, nutritional resources are a focal point of microbial competition (Hibbing et al., 2010). Inhibition of the proliferation of *B. thuringiensis* USDA HD22, *B. cereus* ATCC 11778 (BC45), and *L. monocytogenes* DISTAM MACa1 by the generated active peptide is a drive to maximize nutrient uptake by the *B. cereus* RC6 strain at the expense of other bacteria. Furthermore, a cell producing secreted molecules, like antibiotics, creates an enzymatic protective shell around itself. Within this shell, the cell is also able to simultaneously engage in exploitative competition via its exclusive access to nearby nutrients (Stubbendieck and Straight, 2016).

The comparative genomic analysis revealed misannotated proteins with low homology with *B. cereus* RC6 P1 and P2. Among the 23 *B. cereus* genomes studied, the simultaneous presence of both proteases (essential for the AMP generation) was observed only in 7 genomes, five of them being associated to *B. cereus* isolated from food and the two others having a clinical origin. This finding confirms that our proteases are new and predominantly associated with contaminating *B. cereus*. The secretion of these enzymes, in addition to the metabolic flexibility and toxigenic potential observed in previous studies (Chaabouni et al., 2015), provides an additional strategy for *B. cereus* RC6 within rich microbial communities such as dairy products. We can conclude that a thorough understanding of the bacterial competitive mechanism can help explain the emergence and decline of microbial lineages in natural communities (Hibbing et al., 2010).

## Conclusion

In dairy environments, *B. cereus* live and compete with their neighbors for resources. The consequence of this interaction is the development of new weapons of defense. The *B. cereus* RC6 strain isolated from Tunisian dairy product has developed a special mechanism of defense. This contaminant secrets two new endoproteases that cleave bovine ß-casein, leading to the generation of an AMP that inhibits the proliferation of several species of *Bacillus* and *L. monocytogenes*. A complete understanding and elucidation of the *B. cereus* RC6 strain's strategy to produce AMP from casein is of practical importance, enabling researchers to garner new insights into how contaminant bacteria behave in complex ecosystems such as milk matrices. Furthermore, this knowledge may provide new opportunities for the development of eco-friendly preservatives in dairy products. Such an approach would reduce the use of chemical preservatives and enhance the auto-conservation of milk and dairy products via the use of specific proteolytic enzymes.

## Author contributions

AO, AM, AC, KM, PM, MM, and H-IO conceived and designed the experiments; AO, IC, AM, JL, MB, OM, AN, and PO analyzed the data; PM, AN, ASM, DG, KM, and AC contribution to reagents, material and analysis; AO, IC, AM, JL, MB, PM, H-IO, ASM, MM, DG, KM, and AC manuscript preparation and revision.

### Conflict of interest statement

The authors declare that the research was conducted in the absence of any commercial or financial relationships that could be construed as a potential conflict of interest.

## References

[B1] Abriouel H. Franz C. M. Ben Omar N. Galvez A. (2011). Diversity and applications of Bacillus bacteriocins. FEMS Microbiol. Rev. 35, 201–232. 10.1111/j.1574-6976.2010.00244.x 20695901

[B2] Ahmed A. A. H. Moustafa M. K. Marth E. H. (1983). Incidence of *Bacillus cereus* in milk and some milk products. J. Food Prot. 46, 126–128. 10.4315/0362-028X-46.2.126 30913599

[B3] Altschul S. F. Gish W. Miller W. Myers E. W. Lipman D. J. (1990). Basic local alignment search tool. J. Mol. Biol. 215, 403–410. 10.1016/S0022-2836(05)80360-2 2231712

[B4] Andrusier N. Nussinov R. Wolfson H. J. (2007). FireDock: fast interaction refinement in molecular docking. Proteins 69, 139–159. 10.1002/prot.21495 17598144

[B5] Atanasova J. Ivanova I. (2014). Antibacterial peptides from goat and sheep milk proteins. Biotechnol. Biotechnol. Equip. 24, 1799–1803. 10.2478/V10133-010-0049-8

[B6] Bankevich A. Nurk S. Antipov D. Gurevich A. A. Dvorkin M. Kulikov A. S.. (2012). SPAdes: a new genome assembly algorithm and its applications to single-cell sequencing. J. Comput. Biol. 19, 455–477. 10.1089/cmb.2012.0021 22506599 PMC3342519

[B7] Becker H. Schaller G. von Wiese W. Terplan G. (1994). *Bacillus cereus* in infant foods and dried milk products. Int. J. Food Microbiol. 23, 1–15. 10.1016/0168-1605(94)90218-6 7811567

[B8] Biswas T. Small J. Vandal O. Odaira T. Deng H. Ehrt S.. (2010). Structural insight into serine protease Rv3671c that protects *M. tuberculosis* from oxidative and acidic stress. Structure 18, 1353–1363. 10.1016/j.str.2010.06.017 20947023 PMC2955984

[B9] Bizani D. Dominguez A. P. Brandelli A. (2005). Purification and partial chemical characterization of the antimicrobial peptide cerein 8A. Lett. Appl. Microbiol. 41, 269–273. 10.1111/j.1472-765X.2005.01748.x 16108919

[B10] Boor K. J. Brown D. P. Murphy S. C. Kozlowski S. M. Bandler D. K. (1998). Microbiological and chemical quality of raw milk in New York State. J. Dairy Sci. 81, 1743–1748. 10.3168/jds.S0022-0302(98)75742-X 9684182

[B11] Bottone E. J. (2010). *Bacillus cereus*, a volatile human pathogen. Clin. Microbiol. Rev. 23, 382–398. 10.1128/CMR.00073-09 20375358 PMC2863360

[B12] Bougherraa F. Dilmi-Bourasa A. Baltib R. Przybylskic R. Adouid F. Elhameura H. (2017). Antibacterial activity of new peptide from bovine casein hydrolyzed by a serine metalloprotease of *Lactococcus lactis* subsp lactis BR16. J. Funct. Foods 32, 112–122. 10.1016/j.jff.2017.02.026

[B13] Bullied W. J. Buss T. J. Vessey J. K. (2002). *Bacillus cereus* UW85 inoculation effects on growth, nodulation, and N accumulation in grain legumes: field studies. Can. J. Plant Sci. 82, 291–298. 10.4141/P01-048

[B14] Cao H. Ke T. Liu R. Yu J. Dong C. Cheng M.. (2015). Identification of a novel proline-rich antimicrobial peptide from *Brassica napus*. PLoS ONE 10:e0137414. 10.1371/journal.pone.0137414 26383098 PMC4575134

[B15] Carlin F. Brillard J. Broussolle V. Clavel T. Duport C. Jobin M. (2010). Adaptation of *Bacillus cereus*, an ubiquitous worldwide-distributed foodborne pathogen, to a changing environment. Food Res. Int. 43, 1885–1894. 10.1016/j.foodres.2009.10.024

[B16] Cascales E. Buchanan S. K. Duche D. Kleanthous C. Lloubes R. Postle K.. (2007). Colicin biology. Microbiol. Mol. Biol. Rev. 71, 158–229. 10.1128/MMBR.00036-06 17347522 PMC1847374

[B17] Ceuppens S. Boon N. Uyttendaele M. (2013). Diversity of *Bacillus cereus* group strains is reflected in their broad range of pathogenicity and diverse ecological lifestyles. FEMS Microbiol. Ecol. 84, 433–450. 10.1111/1574-6941.12110 23488744

[B18] Chaabouni I. Barkallah I. Hamdi C. Jouini A. Saidi M. Mahillon J. (2015). Metabolic capacities and toxigenic potential as key drivers of *Bacillus cereus* ubiquity and adaptation. Ann. Microbiol. 65, 975–983. 10.1007/s13213-014-0941-9

[B19] Cherif A. Ettoumi B. Najjari A. Raddadi N. Boudabous A. (2007). Esterase electrophoretic polymorphism of *Bacillus thuringiensis* and *Bacillus cereus* reference strains. Ann. Microbiol. 57, 21–27. 10.1007/BF03175045

[B20] Claudel-Renard C. Chevalet C. Faraut T. Kahn D. (2003). Enzyme-specific profiles for genome annotation: PRIAM. Nucleic Acids Res. 31, 6633–6639. 10.1093/nar/gkg847 14602924 PMC275543

[B21] Dabrowska A. Szoltysik M. Babij K. Pokora M. Zambrowicz A. Chrzanowska J. (2013). Application of asian pumpkin (*Cucurbita ficifolia*) serine proteinase for production of biologically active peptides from casein. Acta Biochim. Pol. 60, 117–122. 23520577

[B22] Davis I. W. Leaver-Fay A. Chen V. B. Block J. N. Kapral G. J. Wang X.. (2007). MolProbity: all-atom contacts and structure validation for proteins and nucleic acids. Nucleic Acids Res. 35, W375–W383. 10.1093/nar/gkm216 17452350 PMC1933162

[B23] Elbarbary H. A. Abdou A. M. Nakamura Y. Park E. Y. Mohamed H. A. Sato K. (2012). Identification of novel antibacterial peptides isolated from a commercially available casein hydrolysate by autofocusing technique. Biofactors 38, 309–315. 10.1002/biof.1023 22539466

[B24] Figueroa-Bossi N. Bossi L. (1999). Inducible prophages contribute to Salmonella virulence in mice. Mol. Microbiol. 33, 167–176. 10.1046/j.1365-2958.1999.01461.x 10411733

[B25] Ghoul M. Mitri S. (2016). The ecology and evolution of microbial competition. Trends Microbiol. 24, 833–845. 10.1016/j.tim.2016.06.011 27546832

[B26] Gohar M. Gilois N. Graveline R. Garreau C. Sanchis V. Lereclus D. (2005). A comparative study of *Bacillus cereus, Bacillus thuringiensis* and *Bacillus anthracis* extracellular proteomes. Proteomics 5, 3696–3711. 10.1002/pmic.200401225 16167365

[B27] Gray E. J. Lee K. D. Souleimanov A. M. Di Falco M. R. Zhou X. Ly A.. (2006). A novel bacteriocin, thuricin 17, produced by plant growth promoting rhizobacteria strain *Bacillus thuringiensis* NEB17: isolation and classification. J. Appl. Microbiol. 100, 545–554. 10.1111/j.1365-2672.2006.02822.x 16478494

[B28] Gurevich A. Saveliev V. Vyahhi N. Tesler G. (2013). QUAST: quality assessment tool for genome assemblies. Bioinformatics 29, 1072–1075. 10.1093/bioinformatics/btt086 23422339 PMC3624806

[B29] Hamdi C. Essanaa J. Sansonno L. Crotti E. Abdi K. Barbouche N.. (2013). Genetic and biochemical diversity of *Paenibacillus* larvae isolated from Tunisian infected honey bee broods. Biomed. Res. Int. 2013:479893. 10.1155/2013/479893 24073406 PMC3774041

[B30] Haque E. Chand R. (2008). Antihypertensive and antimicrobial bioactive peptide from milk proteins. Eur. Food Res. Technol. 227, 7–15. 10.1007/s00217-007-0689-6

[B31] Hayes M. Ross R. P. Fitzgerald G. F. Hill C. Stanton C. (2006). Casein-derived antimicrobial peptides generated by *Lactobacillus acidophilus* DPC6026. Appl. Environ. Microbiol. 72, 2260–2264. 10.1128/AEM.72.3.2260-2264.2006 16517684 PMC1393211

[B32] Hibbing M. E. Fuqua C. Parsek M. R. Peterson S. B. (2010). Bacterial competition: surviving and thriving in the microbial jungle. Nat. Rev. Microbiol. 8, 15–25. 10.1038/nrmicro2259 19946288 PMC2879262

[B33] Huang Y. C. Lin Y. M. Chang T. W. Wu S. J. Lee Y. S. Chang M. D.. (2007). The flexible and clustered lysine residues of human ribonuclease 7 are critical for membrane permeability and antimicrobial activity. J. Biol. Chem. 282, 4626–4633. 10.1074/jbc.M607321200 17150966

[B34] Hyatt D. Chen G. L. Locascio P. F. Land M. L. Larimer F. W. Hauser L. J. (2010). Prodigal: prokaryotic gene recognition and translation initiation site identification. BMC Bioinformatics 11:119. 10.1186/1471-2105-11-119 20211023 PMC2848648

[B35] Ipsen R. Otte J. Lomholt S. B. Qvist K. B. (2000). Standardized reaction times used to describe the mechanism of enzyme-induced gelation in whey protein systems. J. Dairy Res. 67, 403–413. 10.1017/S0022029900004337 11037236

[B36] Jadhav A. A. Khatib S. I. Harale M. A. Gadre S. V. Williamson M. T. (2014). Study of protease enzyme from bacillus species and its application as a contact lens cleanser. Br. Biomed. Bull. 2, 293–302.

[B37] Janštová B. Dračková M. Vorlová L. (2006). Effect of *Bacillus cereus* enzymes on milk quality following ultra high temperature processing. Acta Vet. Brno 75, 601–609. 10.2754/avb200675040601

[B38] Keymer J. E. Galajda P. Muldoon C. Park S. Austin R. H. (2006). Bacterial metapopulations in nanofabricated landscapes. Proc. Natl. Acad. Sci. U.S.A. 103, 17290–17295. 10.1073/pnas.0607971103 17090676 PMC1635019

[B39] Kilcullen K. Teunis A. Popova T. G. Popov S. G. (2016). Cytotoxic potential of *Bacillus cereus* strains ATCC 11778 and 14579 against human lung epithelial cells under microaerobic growth conditions. Front. Microbiol. 7:69. 10.3389/fmicb.2016.00069 26870026 PMC4735842

[B40] Korhonen H. Pihlanto A. (2006). Bioactive peptides: production and functionality. Int. Dairy J. 16, 945–960. 10.1016/j.idairyj.2005.10.012

[B41] Kumari S. Sarkar P. (2016). *Bacillus cereus* hazard and control in industrial dairy processing environment. Food Control 69, 20–29. 10.1016/j.foodcont.2016.04.012

[B42] Lahov E. Regelson W. (1996). Antibacterial and immunostimulating casein-derived substances from milk: casecidin, isracidin peptides. Food Chem. Toxicol. 34, 131–145. 10.1016/0278-6915(95)00097-6 8603791

[B43] LeBlanc J. G. Matar C. Valdez J. C. LeBlanc J. Perdigon G. (2002). Immunomodulating effects of peptidic fractions issued from milk fermented with lactobacillus helveticus. J. Dairy Sci. 85, 2733–2742. 10.3168/jds.S0022-0302(02)74360-9 12487440

[B44] Ledenbach L. H. Marshall R. T. (2009). Microbiological spoilage of dairy products, in Compendium of the Microbiological Spoilage of Foods and Beverages,eds Sperber W. H. Doyle M. P. (New York, NY: Springer New York), 41–67.

[B45] Lehrer R. I. Rosenman M. Harwig S. S. Jackson R. Eisenhauer P. (1991). Ultrasensitive assays for endogenous antimicrobial polypeptides. J. Immunol. Methods 137, 167–173. 10.1016/0022-1759(91)90021-7 1901580

[B46] Li W. Godzik A. (2006). Cd-hit: a fast program for clustering and comparing large sets of protein or nucleotide sequences. Bioinformatics 22, 1658–1659. 10.1093/bioinformatics/btl158 16731699

[B47] López-Expósito I. Quirós A. Amigo L. Recio I. (2007). Casein hydrolysates as a source of antimicrobial, antioxidant and antihypertensive peptides. Lait 87, 241–249. 10.1051/lait:2007019

[B48] Lopez-Fandino R. Olano A. Corzo N. Ramos M. (1993). Proteolysis during storage of UHT milk: differences between whole and skim milk. J. Dairy Res. 60, 339–347. 10.1017/S0022029900027680 8376632

[B49] Majed R. Faille C. Kallassy M. Gohar M. (2016). *Bacillus cereus* biofilms-same, only different. Front. Microbiol. 7:1054. 10.3389/fmicb.2016.01054 27458448 PMC4935679

[B50] Meer R. R. Baker J. Bodyfelt W. Griffiths M. W. (1991). Psychrotrophic *Bacillus* spp. in fluid milk products: a review. J. Food Prot. 54, 969–979. 10.4315/0362-028X-54.12.969 31071827

[B51] Melachouris N. Tuckey S. L. (1968). Properties of a milk-clotting microbial enzyme. J. Dairy Sci. 51, 650–655. 10.3168/jds.S0022-0302(68)87049-3

[B52] Miclo L. Roux E. Genay M. Brusseaux E. Poirson C. Jameh N.. (2012). Variability of hydrolysis of beta-, alphas1-, and alphas2-caseins by 10 strains of *Streptococcus thermophilus* and resulting bioactive peptides. J. Agric. Food Chem. 60, 554–565. 10.1021/jf202176d 22103626

[B53] Mihel J. Sikic M. Tomic S. Jeren B. Vlahovicek K. (2008). PSAIA - protein structure and interaction analyzer. BMC Struct. Biol. 8:21. 10.1186/1472-6807-8-21 18400099 PMC2364630

[B54] Nadell C. D. Xavier J. B. Foster K. R. (2009). The sociobiology of biofilms. FEMS Microbiol. Rev. 33, 206–224. 10.1111/j.1574-6976.2008.00150.x 19067751

[B55] Otvos L. Jr. (2002). The short proline-rich antibacterial peptide family. Cell Mol. Life Sci. 59, 1138–1150. 10.1007/s00018-002-8493-8 12222961 PMC11337521

[B56] Petersen T. N. Brunak S. von Heijne G. Nielsen H. (2011). SignalP 4.0: discriminating signal peptides from transmembrane regions. Nat. Methods 8, 785–786. 10.1038/nmeth.1701 21959131

[B57] Quigley L. O'Sullivan O. Stanton C. Beresford T. P. Ross R. P. Fitzgerald G. F.. (2013). The complex microbiota of raw milk. FEMS Microbiol. Rev. 37, 664–698. 10.1111/1574-6976.12030 23808865

[B58] Raddadi N. Cherif A. Mora D. Brusetti L. Borin S. Boudabous A.. (2005). The autolytic phenotype of the *Bacillus cereus* group. J. Appl. Microbiol. 99, 1070–1081. 10.1111/j.1365-2672.2005.02713.x 16238737

[B59] Rawlings N. D. Barrett A. J. Finn R. (2016). Twenty years of the MEROPS database of proteolytic enzymes, their substrates and inhibitors. Nucleic Acids Res. 44, D343–D350. 10.1093/nar/gkv1118 26527717 PMC4702814

[B60] Riley M. A. Wertz J. E. (2002). Bacteriocins: evolution, ecology, and application. Annu. Rev. Microbiol. 56, 117–137. 10.1146/annurev.micro.56.012302.161024 12142491

[B61] SamarŽija D. Z. S. Pogačić T. (2012). Psychrotrophic bacteria and milk and dairy products quality. Mljekarstvo 62, 77–95.

[B62] Setlow B. Setlow P. (1994). Heat inactivation of *Bacillus subtilis* spores lacking small, acid-soluble spore proteins is accompanied by generation of abasic sites in spore DNA. J. Bacteriol. 176, 2111–2113. 10.1128/jb.176.7.2111-2113.1994 8144480 PMC205320

[B63] Simpson J. T. Wong K. Jackman S. D. Schein J. E. Jones S. J. Birol I. (2009). ABySS: a parallel assembler for short read sequence data. Genome Res. 19, 1117–1123. 10.1101/gr.089532.108 19251739 PMC2694472

[B64] Spano S. Galan J. E. (2012). A Rab32-dependent pathway contributes to *Salmonella* typhi host restriction. Science 338, 960–963. 10.1126/science.1229224 23162001 PMC3693731

[B65] Stubbendieck R. M. Straight P. D. (2016). Multifaceted interfaces of bacterial competition. J. Bacteriol. 198, 2145–2155. 10.1128/JB.00275-16 27246570 PMC4966439

[B66] Stubbendieck R. M. Vargas-Bautista C. Straight P. D. (2016). Bacterial communities: interactions to scale. Front. Microbiol. 7:1234. 10.3389/fmicb.2016.01234 27551280 PMC4976088

[B67] Tannock G. W. (2004). A special fondness for lactobacilli. Appl. Environ. Microbiol. 70, 3189–3194. 10.1128/AEM.70.6.3189-3194.2004 15184111 PMC427720

[B68] Tempelaars M. H. Rodrigues S. Abee T. (2011). Comparative analysis of antimicrobial activities of valinomycin and cereulide, the *Bacillus cereus* emetic toxin. Appl. Environ. Microbiol. 77, 2755–2762. 10.1128/AEM.02671-10 21357430 PMC3126370

[B69] Tewari A. Abdullah S. (2015). *Bacillus cereus* food poisoning: international and Indian perspective. J. Food Sci. Technol. 52, 2500–2511. 10.1007/s13197-014-1344-4 25892750 PMC4397285

[B70] The UniProt C (2017). UniProt: the universal protein knowledgebase. Nucleic Acids Res. 45, D158–D169. 10.1093/nar/gkw1099 27899622 PMC5210571

[B71] Vidal A. M. C. Rossi O. D. Jr. de Abreu I. L. Bürger K. P. Cardoso M. V. Gonçalves A. C. S. (2016). Detection of *Bacillus cereus* isolated during ultra high temperature milk production flowchart through random amplified polymorphic DNA polymerase chain reaction. Ciênc. Rural 46, 286–292. 10.1590/0103-8478cr20141539

[B72] Xu C. Kozlov G. Wong K. Gehring K. Cygler M. (2016). Crystal structure of the *Salmonella typhimurium* effector GtgE. PLoS ONE 11:e0166643. 10.1371/journal.pone.0166643 27923041 PMC5140068

[B73] Yang J. Roy A. Zhang Y. (2013). Protein-ligand binding site recognition using complementary binding-specific substructure comparison and sequence profile alignment. Bioinformatics 29, 2588–2595. 10.1093/bioinformatics/btt447 23975762 PMC3789548

[B74] Yang J. Zhang Y. (2015). Protein structure and function prediction using I-TASSER. Curr. Protoc. Bioinformatics 52, 5.81–5.815. 10.1002/0471250953.bi0508s52 26678386 PMC4871818

[B75] Zhang Y. Skolnick J. (2005). TM-align: a protein structure alignment algorithm based on the TM-score. Nucleic Acids Res. 33, 2302–2309. 10.1093/nar/gki524 15849316 PMC1084323

